# Effects of Single Pill-Based Combination Therapy of Amlodipine and Atorvastatin on Within-Visit Blood Pressure Variability and Parameters of Renal and Vascular Function in Hypertensive Patients with Chronic Kidney Disease

**DOI:** 10.1155/2014/437087

**Published:** 2014-04-08

**Authors:** Kengo Azushima, Kazushi Uneda, Kouichi Tamura, Hiromichi Wakui, Masato Ohsawa, Ryu Kobayashi, Toru Dejima, Tomohiko Kanaoka, Akinobu Maeda, Yoshiyuki Toya, Satoshi Umemura

**Affiliations:** Department of Medical Science and Cardiorenal Medicine, Yokohama City University Graduate School of Medicine, 3-9 Fukuura, Kanazawa-ku, Yokohama 236-0004, Japan

## Abstract

Both strict blood pressure (BP) control and improvements in BP profile such as BP variability are important for suppression of renal deterioration and cardiovascular complication in hypertension and chronic kidney disease (CKD). In the present study, we examined the beneficial effects of the single pill-based combination therapy of amlodipine and atorvastatin on achievement of the target BP and clinic BP profile, as well as markers of vascular and renal damages in twenty hypertensive CKD patients. The combination therapy with amlodipine and atorvastatin for 16 weeks significantly decreased clinic BP, and achievement of target BP control was attained in an average of 45% after the combination therapy in spite of the presence of no achievement at baseline. In addition, the combination therapy significantly decreased the within-visit BP variability. With respect to the effects on renal damage markers, combination therapy with amlodipine and atorvastatin for 16 weeks significantly decreased albuminuria (urine albumin-to-creatinine ratio, 1034 ± 1480 versus 733 ± 1218 mg/g-Cr, *P* < 0.05) without decline in estimated glomerular filtration rate. Concerning parameters of vascular function, the combination therapy significantly improved both brachial-ankle pulse wave velocity (baPWV) and central systolic BP (cSBP) (baPWV, 1903 ± 353 versus 1786 ± 382 cm/s, *P* < 0.05; cSBP, 148 ± 19 versus 129 ± 23 mmHg, *P* < 0.01). Collectively, these results suggest that the combination therapy with amlodipine and atorvastatin may exert additional beneficial effects on renal and vascular damages as well as BP profile in addition to BP lowering in hypertension with CKD.

## 1. Introduction

Chronic kidney disease (CKD) patients are reportedly increasing in number, and cardiovascular complications are the most common cause of death in these patients. Thus, it would be a considerable advance in the management of this condition to elucidate the mechanisms involved in the renal deterioration and the cardiovascular events associated with hypertension complicated by CKD and to identify therapeutic approaches to treat them. Accumulated results of clinical trials also showed that strict control of blood pressure (BP) is essential to prevent target organ damage and to reduce cardiovascular mortality in hypertensive CKD patients [1, 2]. The dihydropyridine calcium channel blocker (CCB) is one of the first-line antihypertensive drugs for most patients with hypertension and is known to exert an efficient BP lowering effect and a strong inhibitory effect on cardiovascular events [3, 4].

In addition, therapies that lower lipid levels also slow the progression of atherosclerosis and reduce morbidity and mortality in patients with hypertension or atherosclerotic disease. The Anglo-Scandinavian Cardiac Outcomes Trial (ASCOT) demonstrated an additive benefit of combined antihypertensive and lipid-lowering therapy on the prevention of cardiovascular complication in high-risk hypertension [5]. Vascular dysfunction, with associated changes in endothelial function and vascular structure, is a risk factor for cardiovascular events through its contribution to the development of atherosclerotic vascular disease. Previous investigations demonstrated improved vascular function and arterial compliance with statins and several antihypertensive drugs such as renin-angiotensin system inhibitors and CCB [6–8]. Both amlodipine and atorvastatin have independently been noted to exert favorable effects on arterial compliance and endothelial dysfunction [8, 9]. This study aimed to examine the beneficial effects of single pill-based combination therapy with amlodipine and atorvastatin on clinic BP profile including within-visit BP variability, a recently emerging marker of linking between kidney and vasculature, and parameters of vascular and renal function in Japanese hypertensive CKD patients who did not achieve the target BP level according to the Japanese Society of Hypertension Guidelines for the Management of Hypertension (JSH2009) [10].

## 2. Materials and Methods

This study was conducted in accordance with the ethical principles of the Declaration of Helsinki and was approved by the Ethics Committees of Yokohama City University Hospital (UMIN 000009045; http://www.umin.ac.jp/ctr/). All of the patients provided written informed consent prior to the start of the study.

### 2.1. Study Participants and Design

Hypertensive patients with CKD who have already been treated with antihypertensive therapy were eligible for the study if they could not achieve the BP goal (clinic systolic BP ≥ 130 mmHg and/or diastolic BP ≥ 80 mmHg), and their low-density lipoprotein (LDL) cholesterol levels were 100 mg/dL or more. CKD was diagnosed by the presence for more than 3 months of albuminuria (urine albumin-to-creatinine ratio, UACR ≥ 30 mg/g-Cr), proteinuria (urine protein-to-creatinine ratio, UPCR ≥ 0.15 g/g-Cr), or estimated glomerular filtration rate (eGFR) < 60 mL/min/1.73 m^2^. We calculated the eGFR using a revised equation for the Japanese population: eGFR (mL/min/1.73 m^2^) = 194 × serum creatinine^−1.094^  × age^−0.287^  × 0.739 (if female) [11]. The exclusion criteria included CKD patients of G5 category, patients who were 19 years old or younger, women who were nursing or pregnant, clinically significant heart disease, stroke, renal artery stenosis, hepatic dysfunction, and known hypersensitivity to any ingredient in the study medications.

After the run-in period, eligible patients were given a single pill of amlodipine/atorvastatin tablet (CADUET, amlodipine besylate/atorvastatin calcium; 2.5/5, 2.5/10, 5/5, and 5/10 mg) for 16 weeks. The starting dose of the single amlodipine/atorvastatin tablet was determined according to each physician's choice, and it was titrated up as needed to achieve the BP and lipid goal (clinic systolic BP < 130 mmHg, diastolic BP < 80 mmHg, and LDL-cholesterol < 100 mg/dL). If patients had already been treated with another calcium channel blocker or statin before the start of the study, they were switched to the single amlodipine/atorvastatin tablet of same dose during the run-in period. If the BP and lipid goal could not be achieved by maximum doses of amlodipine and atorvastatin (10 mg/day and 20 mg/day), another antihypertensive drug and lipid-lowering drug were considered to be added. The doses of other drugs such as oral glucose lowering agents and anticoagulant agents were not changed during the treatment period.

### 2.2. Clinic BP and Within-Visit BP Variability

The clinic BP was measured in sitting position using a calibrated standard mercury sphygmomanometer and the recommended cuff size [12]. Three measurements were taken at 1-minute interval, and their average was regarded as the clinic BP. Within-visit BP variability, which has been reported to be associated with the risk of stroke and cardiovascular risk factors [13, 14], is defined as the within-patient standard deviation (SD) of three measurements of clinic systolic and diastolic BP (within-visit BP variability, SD) and those divided by the each mean clinic BP (within-visit BP variability, CV%).

### 2.3. Central Hemodynamics

The central systolic blood pressure (cSBP) and augmentation index (AI) were measured using an HEM-9000AI (Omron Healthcare, Kyoto, Japan) with an automatic tonometry probe wrapped onto the wrist to record radial waveforms, which were calibrated against the contralateral arm cuff brachial BP taken immediately after tonometry. An algorithm based on a linear regression model was then applied to estimate the cSBP from the “late systolic shoulder” (pSBP2) of the radial pulse waveform, which has been shown to closely agree with the cSBP [15–18]. This device uses the maxima of the “multidimensional derivatives” on the recorded pressure waveforms to detect the first and second inflection points corresponding to the early and late systolic (pSBP2) pressure readings.

### 2.4. Brachial-Ankle Pulse Wave Velocity (baPWV)

The baPWV values were determined by a PP analyzer (model: BP-203RPE2; Omron Healthcare, Kyoto, Japan), as described previously [19–21]. The baPWV values obtained by this method are reported to be significantly correlated with the aortic PWV determined by the catheter method [22].

### 2.5. Laboratory Measurements

Blood and urine sampling were performed in fasted state at baseline and after a period of 16-week treatment, respectively. All parameters were determined by routine methods in the Department of Clinical Chemistry, Yokohama City University School Hospital.

### 2.6. Statistical Analysis

All data were presented as the mean ± SD or as a percentage. For the statistical analysis of the difference between baseline and 16 weeks of treatment, analysis of variance was compared by a paired comparison *t*-test. Univariate and multivariate linear regression analyses were performed to identify the factors affecting the changes in cSBP and UACR. The independent variables entered into the multivariate model were those that were significantly associated in the univariate analyses or were significantly different after a period of 16-week treatment. Analysis was performed using SPSS version 19.0 (IBM Corporation), and a value of *P* < 0.05 was considered statistically significant.

## 3. Results

### 3.1. Baseline Patient Characteristics

Table 1 shows the baseline characteristics of the total 20 hypertensive CKD patients enrolled from September, 2012, to November, 2013. The causes of CKD were hypertensive nephrosclerosis (*n* = 11), diabetic nephropathy (*n* = 6), chronic glomerulonephritis (*n* = 2), and obstructive nephropathy due to urinary tract stones (*n* = 1). None of the enrolled patients discontinued participation in the study. Mean age was 67.7 ± 12.4 years, and the number of males and females was 14 and 6, respectively. Body mass index was 26.8 ± 4.9 kg/m^2^, suggesting that the participants correspond to obese hypertensive patients as a whole. With respect to BP control, clinic BP did not at all achieve the target BP level according to the JSH2009 guideline (clinic SBP/DBP 151 ± 15/81 ± 8 mm Hg) [10].

### 3.2. Effects of Combination Therapy with Amlodipine and Atorvastatin on BP Profile, Glucose and Lipid Metabolism, and Inflammatory and Oxidative Stress Markers

As a whole, the combination therapy with amlodipine and atorvastatin for 16 weeks significantly decreased clinic SBP and DBP (Table 2), and clinic pulse rate was also reduced by the combination therapy (Table 2). In addition, achievement of target BP control, which was defined as BP values less than 130/80 mmHg for CKD patients according to the JSH2009, was attained in an average of 45% after the combination therapy in spite of the presence of no achievement at baseline. Furthermore, the combination therapy significantly decreased the within-visit SBP and DBP variability (BP-SD and BP-CV) (Table 2).

With respect to the glucose and lipid metabolism, the combination therapy with amlodipine and atorvastatin significantly lowered total and LDL-cholesterol levels, without evident changes in parameters of glucose metabolism and inflammation (Table 3). In addition, achievement of the lipid goal, which was defined as LDL-cholesterol level less than 100 mg/dL, was attained in an average of 70% after the combination therapy in spite of the presence of no achievement at baseline. On the other hand, the serum pentosidine level was significantly increased by the combination therapy (Table 3). During a period of 16-week treatment, only two patients who could not achieve the BP goal by the maximum dose of amlodipine (10 mg/day) were additionally administered valsartan 80 mg/day and hydrochlorothiazide 12.5 mg/day, respectively. On the other hand, none of patients was additionally administered lipid-lowering drugs other than atorvastatin. The average doses of amlodipine and atorvastatin were 7.1 ± 2.6 mg/day and 7.0 ± 3.0 mg/day, respectively.

### 3.3. Effects of Combination Therapy with Amlodipine and Atorvastatin on Renal and Vascular Function Parameters

Combination therapy with amlodipine and atorvastatin for 16 weeks significantly decreased UACR (Table 4; UACR, 1034 ± 1480 versus 733 ± 1218 mg/g-Cr, *P* < 0.05). In addition, the reduction of UACR by the combination therapy was not accompanied with decline in eGFR (Table 4; eGFR, 60.4 ± 25.7 versus 58.8 ± 25.6 mL/min/1.73 m^2^). Concerning parameters of vascular function, the combination therapy with amlodipine and atorvastatin for 16 weeks significantly improved both baPWV and cSBP (Table 4; baPWV, 1903 ± 353 versus 1786 ± 382 cm/s, *P* < 0.05; cSBP, 148 ± 19 versus 129 ± 23 mmHg, *P* < 0.01).

### 3.4. Multivariate Regression Analysis for Assessment of Factors Contributing to Improvements in Central BP and Albuminuria

Multivariate regression analysis of the independent variables, which showed significant correlations in the univariate analyses or were significantly different after a period of 16-week treatment, indicated that there were significant associations between the changes in cSBP and those in SBP and total cholesterol and changes in UACR and those in serum creatinine and total cholesterol (Table 5).

## 4. Discussion

The main finding of this study was that the single pill-based administration for combinatorial antihypertensive and LDL cholesterol-lowering therapy with amlodipine and atorvastatin successfully decreased clinic BP so as to improve the achievement of target BP in hypertensive CKD patients already being treated before the start of the combination therapy. In addition, the combination therapy was able to significantly reduce UACR without decrease in eGFR and resulted in significant improvements in vascular function and lipid metabolism. These pleiotropic therapeutic effects by combination therapy with amlodipine and atorvastatin on biomarkers of renal and vascular damage deserve further discussion.

Recent clinical guidelines for hypertensive patients recommend combination therapy such as renin-angiotensin system inhibitors and CCB or diuretics, and, in this study, the combination therapy with amlodipine and atorvastatin was effective for efficient lowering of clinic BP and inhibition of within-visit BP variability in Japanese hypertensive CKD patients. With respect to BP-lowering efficacy of amlodipine, we previously showed that the amlodipine add-on group exerted greater reductions of clinic BP and home BP than the ARB add-on group [23].

Accumulated evidence indicates that renin-angiotensin system inhibitors such as ARB and ACE inhibitors are able to improve albuminuria better than CCB such as amlodipine through the reduction of intraglomerular pressure [24]. However, the combination therapy with amlodipine and atorvastatin exerted a significant reduction in albuminuria in the present study. Of note, in multivariate analysis, the decrease in total-cholesterol level was an independent contributing factor to the decrease in UACR. This result suggests that statin therapy may have a favorable effect on albuminuria and is confirmed with previous studies [25–28].

A previous study showed that the decreases in BP significantly contributed to the decreases in albuminuria by CCB in hypertensive and CKD patients [23, 29]. Although analysis of patient characteristics at baseline unexpectedly revealed that all participants were already treated with CCB in spite of only 30% of the participants being treated with statin, the combination therapy with amlodipine and atorvastatin for 16 weeks succeeded to efficiently suppress albuminuria, irrespective of preceding medication, without further decline in eGFR. This is likely to be an important advantage of the combination therapy with amlodipine and atorvastatin, since several recent epidemiological studies and intervention trials demonstrated that efficient reduction of albuminuria with preserved eGFR is important to inhibit the progression of CKD and to prevent the development of cardiovascular complication [30–32].

The combination therapy with amlodipine and atorvastatin exhibited a significant improvement in vascular functional parameters such as baPWV and cSBP in this study. A previous study showed that the add-on amlodipine therapy had benefits in terms of the vascular function and vascular structure of hypertensive patients already treated with renin-angiotensin inhibitors, which were independent of its depressor effects but with a concomitant decrease in ambulatory BP variability [33]. Also, these results are consistent with a previous study showing that coadministered amlodipine and atorvastatin produced early improvements in arterial wall compliance in hypertensive patients with dyslipidemia [34]. Since statins may exert their vascular beneficial effects by inhibiting small GTPase protein synthesis, such as Ras and Rho kinase [35, 36], the combination therapy of amlodipine and atorvastatin may have an additive beneficial effect on the vasculature so as to improve vascular atherosclerotic changes [8, 9].

Recently, parameters of BP variability obtained by clinic BP measurement are suggested to be associated with target organ damage including CKD and cardiovascular complication [37–39]. Variation in BP that is captured at sequential office visits is called visit-to-visit variability, and BP variation that is captured within each office visit is called within-visit BP variability. Accumulated evidence has indicated that greater degrees of clinic BP variability such as visit-to-visit BP variability and within-visit BP variability are associated with renal deterioration and cardiovascular complication [14, 37–40]. Almost all reported results showed that these parameters of BP variability, such as clinic BP variability and home-measured BP variability in addition to ambulatory BP variability, reflect organ damages and are potential predictors of cardiovascular events [37, 41, 42]. Furthermore, these analyses also displayed that CCB is the most effective drug class for reduction of BP variability [43].

A recent study showed that alteration of vascular function is an important factor in the correlation between clinic BP variability and cardiovascular disease [44], and clinic BP variability is recently suggested to correlate significantly with renal function and renal arteriosclerotic change as an emerging candidate of linking factor between vascular alteration and renal damage [38]. The results of the present study demonstrated that the combination therapy with amlodipine and atorvastatin significantly decreased clinic BP and its variability concomitant with improvements in parameters of vascular and renal function. In the present study, there was no significant relationship between the improvement in lipid metabolism and that in clinic BP and within-visit BP variability on univariate analysis (data not shown). Therefore, the association between statin therapy and BP or BP variability is still to be determined in the future study.

In the present study, pulse rate was significantly decreased by combination therapy of amlodipine and atorvastatin in spite of BP reduction. It has been reported that amlodipine tends to increase pulse rate via the reflex stimulation of sympathetic nervous system [45]. On the other hand, there are several previous studies suggesting that statins can reduce sympathetic activity, thereby resulting in pulse rate reduction [46, 47]. Thus, the decrease in pulse rate may be caused by concomitant statin therapy. However, since there is no direct data which support this possibility in the present study, further investigation is needed to elucidate this issue.

There are several limitations in the present study. Firstly, the present study is not randomized parallel-group control trial, and the numbers are small. Secondly, the patient's adherence was not examined in the present study. Since there are several previous studies suggesting that single-pill amlodipine/atorvastatin rather than 2-pill regimen may reduce cardiovascular events by improving the patient's adherence [48, 49], the improving effect of single-pill amlodipine/atorvastatin therapy on patient's adherence may have an influence on the results of this study. Further studies are needed to estimate the true long-term advantage of the amlodipine/atorvastatin combination in lowering blood pressure and improve vascular damage.

## 5. Conclusions

In summary, the results of the present study suggest that the combination therapy with amlodipine and atorvastatin may exert additional beneficial effects on renal and vascular damages as well as BP profile in addition to BP lowering in hypertension with CKD.

## Figures and Tables

**Table 1:** Baseline characteristics.

Variables	Mean ± SD or %
Age (y)	67.7 ± 12.4
Sex (male/female)	14/6
Body mass index (kg/m^2^)	26.8 ± 4.9
Alcohol (%)	40
Smoking (%)	20
Diabetes mellitus (%)	40
Dyslipidemia (%)	85
Previous cardiovascular disease (%)	20
Clinic blood pressure	
SBP (mmHg)	151 ± 15
DBP (mmHg)	81 ± 8
Glucose-lipid metabolism	
Fasting plasma glucose (mg/dL)	130 ± 51
Glycated hemoglobin (%)	6.1 ± 0.8
Total cholesterol (mg/dL)	230 ± 33
LDL-cholesterol (mg/dL)	143 ± 27
HDL-cholesterol (mg/dL)	63 ± 21
Triglycerides (mg/dL)	195 ± 140
Renal function	
Serum creatinine (mg/dL)	1.04 ± 0.44
eGFR (mL/min/1.73 m^2^)	60.4 ± 25.8
UACR (mg/g-Cr)	1034 ± 1480
Serum cystatin C (mg/L)	1.30 ± 0.45
Inflammatory and oxidative stress markers	
hs-CRP (mg/dL)	0.18 ± 0.24
Serum pentosidine (*μ*g/mL)	0.036 ± 0.013
Medication	
Statin (%)	30
Calcium channel blockers (%)	100
RAS inhibitors (%)	85

Values are means ± SD. SBP: systolic blood pressure; DBP: diastolic blood pressure; eGFR: estimated glomerular filtration rate; UACR: urine albumin-to-creatinine ratio; CRP: C-reactive protein; RAS: renin-angiotensin system.

**Table 2:** Effects of single-pill amlodipine/atorvastatin-based therapy on clinic blood pressure profile.

	Baseline	Week 16
Clinic blood pressure		
SBP (mmHg)	151 ± 15	131 ± 11**
SBP-SD	6.1 ± 2.5	3.1 ± 2.2**
SBP-CV (%)	4.1 ± 1.6	2.4 ± 1.8**
DBP (mmHg)	81 ± 8	74 ± 10**
DBP-SD	4.6 ± 2.6	2.3 ± 1.7**
DBP-CV (%)	5.6 ± 3.1	3.1 ± 2.3*
Pulse rate (beat/min)	74 ± 7	69 ± 7**

Values are means ± SD. SBP: systolic blood pressure; DBP: diastolic blood pressure. *P < 0.05, **P < 0.01.

**Table 3:** Effects of single-pill amlodipine/atorvastatin-based therapy on glucose-lipid metabolism and inflammatory and oxidative stress markers.

	Baseline	Week 16
Glucose-lipid metabolism		
Fasting plasma glucose (mg/dL)	130 ± 51	127 ± 56
Glycated hemoglobin (%)	6.1 ± 0.8	6.2 ± 0.9
Total cholesterol (mg/dL)	230 ± 33	180 ± 37**
LDL-cholesterol (mg/dL)	143 ± 27	97 ± 30**
HDL-cholesterol (mg/dL)	63 ± 21	61 ± 19
Triglycerides (mg/dL)	195 ± 140	180 ± 134
Inflammatory and oxidative stress markers		
hs-CRP (mg/dL)	0.18 ± 0.24	0.09 ± 0.10
Serum pentosidine (*μ*g/mL)	0.036 ± 0.013	0.046 ± 0.018**

Values are means ± SD. CRP: C-reactive protein. **P < 0.01.

**Table 4:** Effects of single-pill amlodipine/atorvastatin-based therapy on renal and vascular functions.

	Baseline	Week 16
Renal function		
Serum creatinine (mg/dL)	1.04 ± 0.44	1.09 ± 0.50
eGFR (mL/min/1.73 m^2^)	60.4 ± 25.7	58.8 ± 25.6
UACR (mg/g-Cr)	1034 ± 1480	733 ± 1218*
Serum cystatin C (mg/L)	1.30 ± 0.45	1.31 ± 0.49
Vascular function		
baPWV (cm/s)	1903 ± 353	1786 ± 382*
AI (%)	84 ± 15	81 ± 14
cSBP (mmHg)	148 ± 19	129 ± 23**

Values are means ± SD. eGFR: estimated glomerular filtration rate; UACR: urine albumin-to-creatinine ratio; baPWV: brachial-ankle pulse wave velocity; AI: augmentation index; cSBP: central systolic blood pressure.

*P < 0.05, **P < 0.01.

**Table 5:** Multivariate linear regression analyses of factors associated with changes in cSBP and UACR.

Variables	*β*	*P* value
Change in cSBP (mmHg)		
Change in SBP (mmHg)	0.654	0.001
Change in total cholesterol (mg/dL)	0.439	0.020
(Model *R* ^2^ = 0.679)		
Change in UACR (mg/g-Cr)		
Change in SBP (mmHg)	−0.027	0.885
Change in serum creatinine (mg/dL)	−0.729	0.002
Change in total cholesterol (mg/dL)	0.418	0.034
(Model *R* ^2^ = 0.643)		

*R* ^2^: coefficient of determination. cSBP: central systolic blood pressure; UACR: urine albumin-to-creatinine ratio. These values are adjusted by age and sex.

## References

[1] Casas JP, Chua W, Loukogeorgakis S, Vallance P, Smeeth L, Hingorani AD (2005). Effect of inhibitors of the renin-angiotensin system and other antihypertensive drugs on renal outcomes: systematic review and meta-analysis. *The Lancet*.

[2] Ninomiya T, Perkovic V, Turnbull F (2013). Blood pressure lowering and major cardiovascular events in people with and without chronic kidney disease: meta-analysis of randomised controlled trials. *British Medical Journal*.

[3] Berl T, Hunsicker LG, Lewis JB (2003). Cardiovascular outcomes in the irbesartan diabetic nephropathy trial of patients with type 2 diabetes and overt nephropathy. *Annals of Internal Medicine*.

[4] Julius S, Kjeldsen SE, Weber M (2004). Outcomes in hypertensive patients at high cardiovascular risk treated with regimens based on valsartan or amlodipine: the value randomised trial. *The Lancet*.

[5] Dahlöf B, Sever PS, Poulter NR (2005). Prevention of cardiovascular events with an antihypertensive regimen of amlodipine adding perindopril as required versus atenolol adding bendroflumethiazide as required, in the Anglo-Scandinavian Cardiac Outcomes Trial-Blood Pressure Lowering Arm (ASCOT-BPLA): a multicentre randomised controlled trial. *The Lancet*.

[6] Higashi Y, Sasaki S, Nakagawa K (2000). A comparison of angiotensin-converting enzyme inhibitors, calcium antagonists, beta-blockers and diuretic agents on reactive hyperemia in patients with essential hypertension: a multicenter study. *Journal of the American College of Cardiology*.

[7] Nazzaro P, Manzari M, Merlo M (1999). Distinct and combined vascular effects of ACE blockade and HMG-CoA reductase inhibition in hypertensive subjects. *Hypertension*.

[8] Leibovitz E, Beniashvili M, Zimlichman R, Freiman A, Shargorodsky M, Gavish D (2003). Treatment with amlodipine and atorvastatin have additive effect in improvement of arterial compliance in hypertensive hyperlipidemic patients. *American Journal of Hypertension*.

[9] Zhou MS, Tian R, Jaimes EA, Raij L (2014). Combination therapy of amlodipine and atorvastatin has more beneficial vascular effects than monotherapy in salt-sensitive hypertension. *American Journal of Hypertension*.

[10] Ogihara T, Kikuchi K, Matsuoka H (2009). The Japanese society of hypertension guidelines for the management of hypertension (JSH 2009). *Hypertension Research*.

[11] Matsuo S, Imai E, Horio M (2009). Revised equations for estimated GFR from serum creatinine in Japan. *American Journal of Kidney Diseases*.

[12] Pickering TG, Hall JE, Appel LJ (2005). Recommendations for blood pressure measurement in humans: an AHA scientific statement from the Council on High Blood Pressure Research Professional and Public Education Subcommittee. *Journal of Clinical Hypertension*.

[13] Rothwell PM, Howard SC, Dolan E (2010). Effects of *β* blockers and calcium-channel blockers on within-individual variability in blood pressure and risk of stroke. *The Lancet Neurology*.

[14] Shin JH, Shin J, Kim BK (2013). Within-visit blood pressure variability: relevant factors in the general population. *Journal of Human Hypertension*.

[15] Takazawa K, Kobayashi H, Shindo N, Tanaka N, Yamashina A (2007). Relationship between radial and central aeterial pulse wave and evaluation of central aortic pressure using the radial arterial pulse wave. *Hypertension Research*.

[16] Kips JG, Schutte AE, Vermeersch SJ (2011). Comparison of central pressure estimates obtained from SphygmoCor, Omron HEM-9000AI and carotid applanation tonometry. *Journal of Hypertension*.

[17] Rezai M-R, Goudot G, Winters C, Finn JD, Wu FC, Cruickshank JK (2011). Calibration mode influences central blood pressure differences between SphygmoCor and two newer devices, the Arteriograph and Omron HEM-9000. *Hypertension Research*.

[18] Tomiyama H, Odaira M, Matsumoto C (2011). Effects of moderate-to-severe impairment of the estimated glomerular filtration rate and of proteinuria on the central hemodynamics and arterial stiffness in middle-aged healthy Japanese men. *International Journal of Nephrology*.

[19] Mitsuhashi H, Tamura K, Yamauchi J (2009). Effect of losartan on ambulatory short-term blood pressure variability and cardiovascular remodeling in hypertensive patients on hemodialysis. *Atherosclerosis*.

[20] Masuda S-I, Tamura K, Wakui H (2009). Effects of angiotensin II type 1 receptor blocker on ambulatory blood pressure variability in hypertensive patients with overt diabetic nephropathy. *Hypertension Research*.

[21] Kanaoka T, Tamura K, Ohsawa M (2012). Effects of aliskiren-based therapy on ambulatory blood pressure profile, central hemodynamics, and arterial stiffness in nondiabetic mild to moderate hypertensive patients. *Journal of Clinical Hypertension*.

[22] Yamashina A, Tomiyama H, Takeda K (2002). Validity, reproducibility, and clinical significance of noninvasive brachial-ankle pulse wave velocity measurement. *Hypertension Research*.

[23] Maeda A, Tamura K, Kanaoka T (2012). Combination therapy of angiotensin II receptor blocker and calcium channel blocker exerts pleiotropic therapeutic effects in addition to blood pressure lowering: amlodipine and candesartan trial in Yokohama (ACTY). *Clinical and Experimental Hypertension*.

[24] Viberti G, Wheeldon NM (2002). Microalbuminuria reduction with valsartan in patients with type 2 diabetes mellitus: a blood pressure-independent effect. *Circulation*.

[25] Nitta K (2012). Clinical assessment and management of dyslipidemia in patients with chronic kidney disease. *Clinical and Experimental Nephrology*.

[26] Douglas K, O’Malley PG, Jackson JL (2006). Meta-analysis: the effect of statins on albuminuria. *Annals of Internal Medicine*.

[27] Strippoli GFM, Navaneethan SD, Johnson DW (2008). Effects of statins in patients with chronic kidney disease: meta-analysis and meta-regression of randomised controlled trials. *British Medical Journal*.

[28] Sandhu S, Wiebe N, Fried LF, Tonelli M (2006). Statins for improving renal outcomes: a meta-analysis. *Journal of the American Society of Nephrology*.

[29] Kaneshiro Y, Ichihara A, Sakoda M, Kurauchi-Mito A, Kinouchi K, Itoh H (2009). Add-on benefits of amlodipine and thiazide in nondiabetic chronic kidney disease stage 1/2 patients treated with valsartan. *Kidney and Blood Pressure Research*.

[30] Bakris GL, Sarafidis PA, Weir MR (2010). Renal outcomes with different fixed-dose combination therapies in patients with hypertension at high risk for cardiovascular events (ACCOMPLISH): a prespecified secondary analysis of a randomised controlled trial. *The Lancet*.

[31] Matsushita K, van der Velde M, Astor BC (2010). Association of estimated glomerular filtration rate and albuminuria with all-cause and cardiovascular mortality in general population cohorts: a collaborative meta-analysis. *The Lancet*.

[32] Herzog CA, Asinger RW, Berger AK (2011). Cardiovascular disease in chronic kidney disease. A clinical update from kidney disease improving global outcomes (KDIGO). *Kidney International*.

[33] Ichihara A, Kaneshiro Y, Sakoda M, Takemitsu T, Itoh H (2007). Add-on amlodipine improves arterial function and structure in hypertensive patients treated with an angiotensin receptor blocker. *Journal of Cardiovascular Pharmacology*.

[34] Cohn JN, Wilson DJ, Neutel J (2009). Coadministered amlodipine and atorvastatin produces early improvements in arterial wall compliance in hypertensive patients with dyslipidemia. *American Journal of Hypertension*.

[35] Rikitake Y, Liao JK (2005). Rho GTPases, statins, and nitric oxide. *Circulation Research*.

[36] Ito D, Ito O, Mori N (2010). Atorvastatin upregulates nitric oxide synthases with Rho-kinase inhibition and Akt activation in the kidney of spontaneously hypertensive rats. *Journal of Hypertension*.

[37] Rothwell PM, Howard SC, Dolan E (2010). Prognostic significance of visit-to-visit variability, maximum systolic blood pressure, and episodic hypertension. *The Lancet*.

[38] Kawai T, Ohishi M, Kamide K (2012). The impact of visit-to-visit variability in blood pressure on renal function. *Hypertension Research*.

[39] Shimbo D, Newman JD, Aragaki AK (2012). Association between annual visit-to-visit blood pressure variability and stroke in postmenopausal women: data from the women’s health initiative. *Hypertension*.

[40] McMullan CJ, Bakris GL, Phillips RA, Forman JP (2013). Association of BP variability with mortality among African Americans with CKD. *Clinical Journal of the American Society of Nephrology*.

[41] Tamura K, Kanaoka T, Ohsawa M (2011). Emerging concept of anti-hypertensive therapy based on ambulatory blood pressure profile in chronic kidney disease. *American Journal of Cardiovascular Disease*.

[42] Leoncini G, Viazzi F, Storace G, Deferrari G, Pontremoli R (2013). Blood pressure variability and multiple organ damage in primary hypertension. *Journal of Human Hypertension*.

[43] Webb AJ, Fischer U, Mehta Z, Rothwell PM (2010). Effects of antihypertensive-drug class on interindividual variation in blood pressure and risk of stroke: a systematic review and meta-analysis. *The Lancet*.

[44] Kawai T, Ohishi M, Ito N (2013). Alteration of vascular function is an important factor in the correlation between visit-to-visit blood pressure variability and cardiovascular disease. *Journal of Hypertension*.

[45] Toal CB, Meredith PA, Elliott HL (2012). Long-acting dihydropyridine calcium-channel blockers and sympathetic nervous system activity in hypertension: a literature review comparing amlodipine and nifedipine GITS. *Blood Press*.

[46] Siddiqi L, Joles JA, Oey PL, Blankestijn PJ (2011). Atorvastatin reduces sympathetic activity in patients with chronic kidney disease. *Journal of Hypertension*.

[47] Lewandowski J, Siński M, Bidiuk J (2010). Simvastatin reduces sympathetic activity in men with hypertension and hypercholesterolemia. *Hypertension Research*.

[48] Chapman RH, Yeaw J, Roberts CS (2010). Association between adherence to calcium-channel blocker and statin medications and likelihood of cardiovascular events among US managed care enrollees. *BMC Cardiovascular Disorders*.

[49] Patel BV, Leslie RS, Thiebaud P (2008). Adherence with single-pill amlodipine/atorvastatin versus a two-pill regimen. *Vascular Health and Risk Management*.

